# Batista Procedure with the Aid of Intraoperative Epicardial Echocardiography

**DOI:** 10.21470/1678-9741-2019-0298

**Published:** 2020

**Authors:** Ken Nakamura, Tetsuro Uchida, Azumi Hamasaki, Mitsuaki Sadahiro

**Affiliations:** 1 Department of Cardiovascular Surgery, Nihonkai General Hospital, Yamagata, Sakata, Japan.; 2 Department of Surgery II, Yamagata University Faculty of Medicine, Yamagata, Japan.

**Keywords:** Dilated Cardiomyopathy, Papillary Muscles, Natriuretic Peptide, Brain. Heart Ventricles, Heart Failure, Echocardiography

## Abstract

**Introduction:**

The number of cases for which the Batista procedure is indicated is small, but some patients with appropriate indication can achieve good therapeutic results.

**Objective:**

To avoid incorrect left ventricular incision and obtain good surgical results in patients with dilated cardiomyopathy suitable for partial left ventriculectomy, we employed intraoperative direct echocardiography to determine the exact extent and position of the myocardial incision, even for surgeons who are not very experienced with the Batista procedure.

**Methods:**

A 72-year-old man with dilated cardiomyopathy underwent the Batista procedure with the aid of epicardial echocardiography to confirm the location of both the papillary muscles and the diseased myocardium.

**Results:**

We were able to accurately perform left ventricular incision and remove the diseased lateral ventricular wall. Two years later, the patient had no symptoms of heart failure, and his brain natriuretic peptide (BNP) level decreased from 1155 to 49.3 pg/mL.

**Conclusions:**

We believe that the use of intraoperative echocardiography may have the potential to make the Batista procedure less technically demanding and more reproducible for surgeons with little experience in the procedure.

**Table:** 

Abbreviations, acronyms & symbols	
BNP	= Brain natriuretic peptide
CI	= Cardiac index
CO	= Cardiac output
CT	= Computed tomography
IABP	= Intra-aortic balloon pump
IVSd	= Interventricular septal wall thickness at end diastole
LV	= Left ventricular
LVEDD	= Left ventricular end-diastolic dimension
LVESD	= Left ventricular end-systolic dimension
LVESV	= Left ventricular end-systolic volume
LVESVI	= Left ventricular end-systolic volume index
LVPWd	= Left ventricular posterior wall thickness at end diastole
MIBI	= Methoxy isobutyl isonitrile
MR	= Mitral regurgitation
TR	= Tricuspid regurgitation

## INTRODUCTION

Although there are many reports on the Batista procedure, few have shown that it is effective and associated with a good long-term prognosis, so it is not currently performed at many facilities. Few patients have lesion confined to the posterolateral side of the myocardium, and few surgeons perform Batista procedures according to strict criteria.

However, some patients can obtain good therapeutic results, as reported by Batista et al.^[1]^, and if they are appropriate candidates for treatment, this procedure may be an effective option. There may be some patients who need this surgery to improve their heart failure.

We experienced a case in which intraoperative epicardial echocardiography was used to confirm the location of both the papillary muscles and the diseased myocardium, and it was possible to plan the resection site before surgery. This method may be an effective intraoperative diagnostic tool as an aid to surgeons performing the Batista operation who do not have much experience with the procedure. We report our experience in this article.

### Clinical Data

Our patient was a 72-year-old man who had been followed for 4 years at an outside hospital for severe chronic systolic heart failure due to dilated cardiomyopathy and mitral regurgitation (MR). He had been diagnosed with atrial fibrillation four years ago, but the exact time of onset was unknown. With medical management (carvedilol 5 mg, enalapril maleate 5 mg, telmisartan 40 mg, furosemide 20 mg, metildigoxin 0.05 mg, warfarin 2 mg) alone, his disease had progressed, leading to exertional dyspnea. From these findings, our policy was to treat with surgery. On admission to our institution, his height was 153.5 cm, body weight 59.1 kg, blood pressure 98/64 mmHg, heart rate 64 bpm and the rhythm was atrial fibrillation. Electrocardiography revealed atrial fibrillation without f-wave and no left bundle branch block. Echocardiography showed severe MR resulting from tethering and moderate tricuspid regurgitation (TR). Left ventricular (LV) end-diastolic and end-systolic dimensions (LVEDD/LVESD) were 75/66 mm, left ventricular volumetry were 341/225 mL, interventricular septal wall thickness in the end diastole (IVSd) was 11 mm, left ventricular posterior wall thickness in the end diastole (LVPWd) was 5 mm, interpapillary muscle distance was 39 mm and the LV ejection fraction was 28% (modified Simpson’s method). Peak early diastolic filling velocity (E-wave), peak late diastolic filling velocity (A-wave) and their ratio (E/A) was 1.37. The E/e’ (the ratio between early mitral inflow velocity and mitral annular early diastolic velocity) was 14.43. The LV wall showed global hypokinesia and inferoposterior dyskinesia. The chest X-ray revealed bilateral pulmonary congestion. The chest thoracic ratio was 72%. His preoperative BNP was 1155 pg/mL. Coronary computed tomography (CT) revealed no vascular stenoses. Cardiac catheterization showed left ventricular end-systolic volume (LVESV) 267.6 mL, left ventricular end-diastolic volume (LVEDV) 395.2 mL, left ventricular end-systolic volume index (LVESVI) 180 mL/m^2^, cardiac output (CO) 2.2 L/minute, cardiac index (CI) 1.5 L/minute/m^2^. Single-photon emission computed tomography image of exercise 99mTc-methoxy isobutyl isonitrile (MIBI) scintigraphy revealed defect of concentration in the posterior wall and myocardial infarction tracer showed a negative scintigram.

### Surgical Plan

**Fig. 1:**
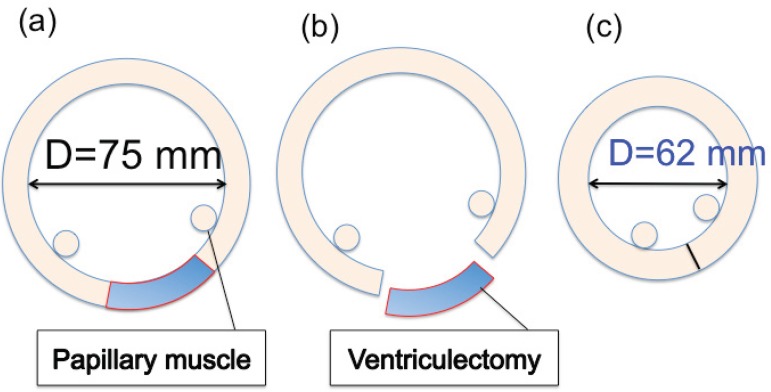
Mathematical modeling of posterior ventricular restoration. Preoperative LVEDD=75 mm. To establish a postoperative LVEDD of 62 mm, 40 mm of ventricular tissue needed to be removed. LVDD=left ventricular end-diastolic diameter

The patient’s dilated cardiomyopathy was due to global hypokinesia and lateral wall dyskinesia. We thus decided to perform the Batista procedure. We did not choose maze procedure and cardiac resynchronization therapy because both were thought to be not effective. To establish a postoperative LVEDD of 62 mm, 40 mm of ventricular tissue needed to be removed (Figure 1). As a practical matter, we needed an incision line of less than 40 mm to establish the marginal area of the suture zone.

### Surgery

Under local anesthesia, an intra-aortic balloon pump (IABP) was inserted into the left femoral artery. The IABP was initiated before the introduction of general anesthesia. The procedure was performed on-pump, with a beating heart and without aortic cross-clamp (Video1). The anterior and posterior pupillary muscles were visualized with epicardial echocardiography (SonoSite S Series, Fujifilm, Washington) (Video 2). The posterior wall was noted to be thin in the region between the posterior descending artery and the obtuse marginal artery. A wedge resection was performed under echocardiogram guidance. A deep and wide mattress suture was performed with 3-0 hexafluoropropylene in an interrupted and continuous fashion to exclude the posterolateral dyskinetic lesion of the left ventricle. The patient was also noted to have moderate-to-severe mitral and tricuspid regurgitation, so mitral valve repair with Physio II ring 26 mm (Edwards Lifesciences Corporation, Irvine, CA) and tricuspid annuloplasty with MC332 mm (Edwards Lifesciences Corporation, Irvine, CA) were performed (Video 3).

**Video 1:**
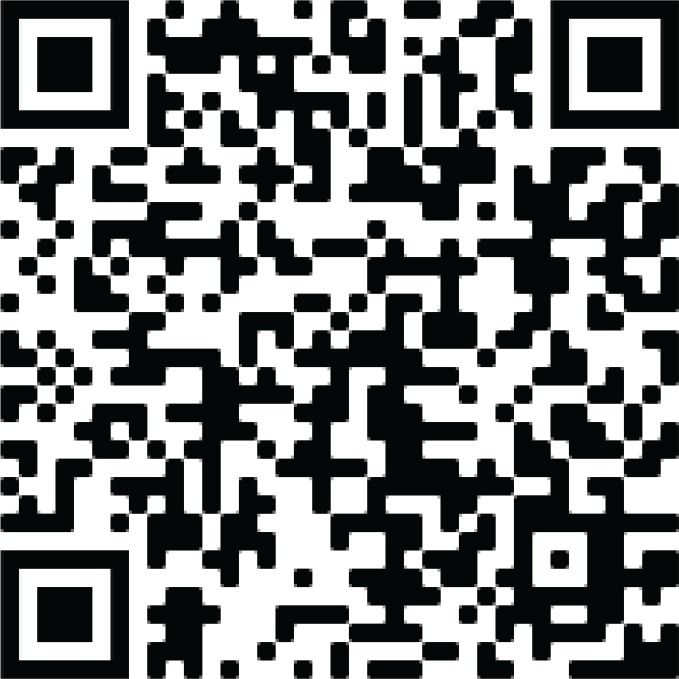
Intraoperative diagnosis with using cardiopulmonary bypass.

**Video 2:**
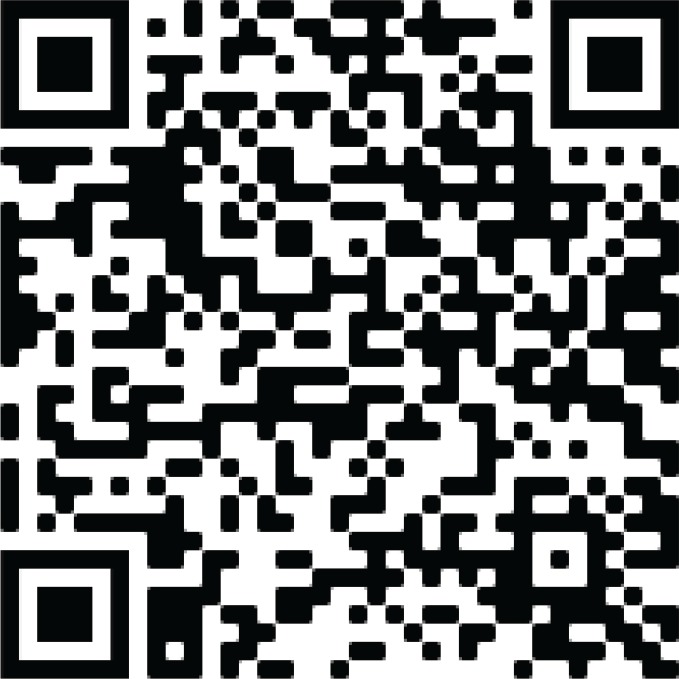
Epicardial echocardiography.

**Video3:**
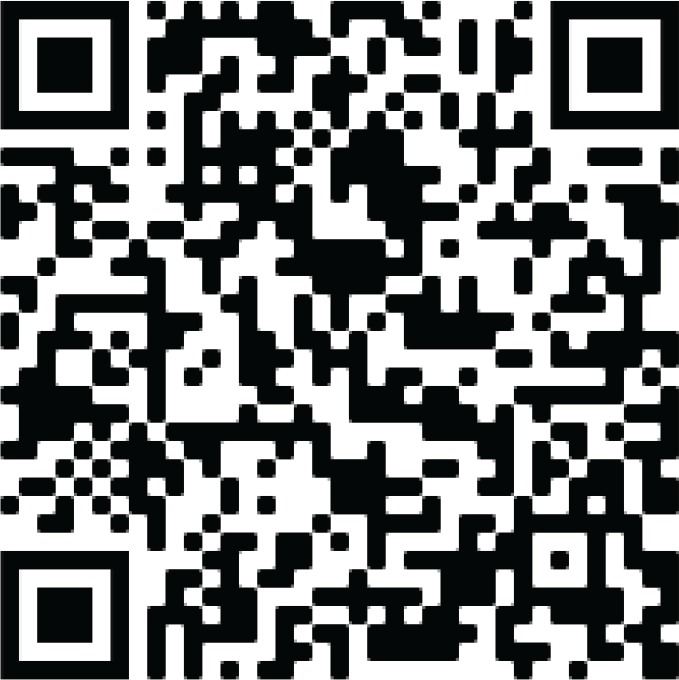
Performing Partial Left Ventriculectomy.

### Postoperative Course

Postoperative echocardiography revealed LVEDD/LVESD of 62/43 mm and LV ejection fraction of 30% (modified Simpson’s method). Mitral regurgitation was not observed (tethering was improving). Three weeks after surgery, the patient was discharged from our hospital without further complication. Histopathological findings revealed the presence of abnormal nuclei (swollen nuclei with an altered structure surrounded by a halo) and loss of striation. Fibrosis was characteristically interstitial and began to surround and isolate individual myocytes. There was no contradiction as a finding of dilated cardiomyopathy.

**Fig. 2:**
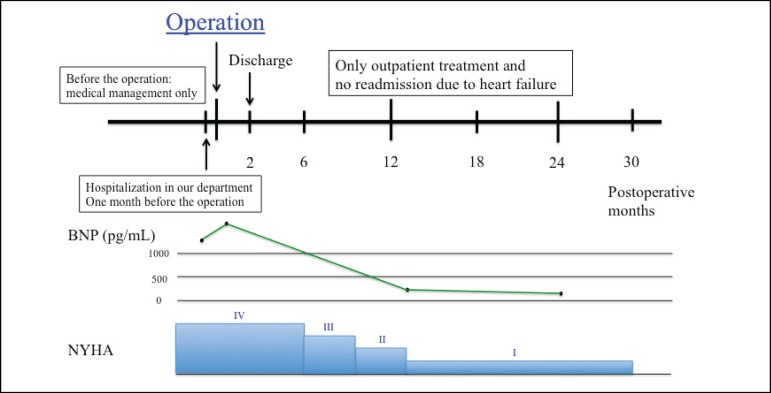
Timeline. On postoperative day (POD) 4, the patient presented abdominal distension. Follow-up computed tomography suggested a segmental lack of enhancement in the intestinal wall without superior mesenteric artery (SMA) occlusion. The patient was diagnosed with nonocclusive mesenteric ischemia (NOMI). We performed selective mesenteric angiography and transcatheter prostaglandin infusion via SMA. Seventeen days after treatment, mesenteric blood flow improved and enteral nutrition was resumed. The patient was discharged from the hospital 2 months after surgery. His outpatient course was good, and his heart failure symptoms gradually improved. BNP=brain natriuretic peptide; NYHA=New York Heart Association

**Fig. 3:**
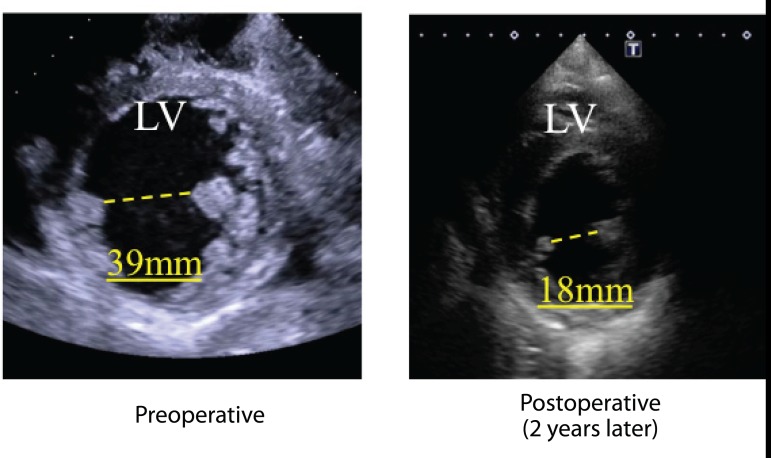
Pre and postoperative transthoracic echocardiography. In the short axis view, the anterior and posterior papillary muscle distance (interpapillary muscle distance) was 39 mm in preoperative echocardiography. Two years later, interpapillary muscle distance was 18 mm.

Two years later, the patient had no symptom and his BNP level decreased from 1155 to 49.3 pg/mL (Figure 2). Echocardiography showed LVEDD/LVESD of 67/41 mm, left ventricular volumetry were 176/138 mL, E/A was 1.22, E/e’ was 1.3 and an LV ejection fraction of 31% (modified Simpson’s method). Mitral regurgitation was trivial and tricuspid regurgitation was none. In the short axis view, the anterior and posterior papillary muscle was approximate compared to preoperative echocardiography (interpapillary muscle distance was reduced from 39 mm to 18 mm) (Figure 3).

## DISCUSSION

The Batista operation has been shown to improve survival in carefully selected patient populations^[1,2]^ The procedure improves LV function by decreasing LV diameter via excision of the diseased LV free wall. Here, we report the use of the Batista operation in a patient with end-stage cardiomyopathy, with dyskinesia of the posterolateral wall between the anterior and posterior papillary muscles, and a dilated LVEDD ≥75 mm. For this patient, we aimed for a postoperative LVEDD of 65 mm. The Batista operation consisted of a wide excision of the posterolateral wall between the anterior and posterior papillary muscles.

The choice of LV excision or exclusion site is difficult, even for experienced cardiac surgeons. We used direct intraoperative echocardiography to select the site of LV excision. Echocardiography allowed us to detect the diseased LV wall region and the location of the anterior and posterior papillary muscles. Isomura^[3]^ introduced the echo-guided volume reduction test and evaluated its effects on surgery for dilated cardiomyopathy. Here, we report the first use of direct intraoperative echocardiography to select the location of the surgical incision line.

Although in many countries the Batista operation has been largely abandoned following a negative report from the Cleveland Clinic^[4]^, suitable patient selection and proper surgical technique may improve LV function and provide effective treatment^[5]^. Isomura et al. recently noted that surgeons will be able to select several options for the treatment of congestive heart failure; this includes the use of mechanical support devices and the use of implantable ventricular assist devices as target therapy^[6]^. Posterior cardiac restoration was one of these treatments, and the most important issue was understanding the advantages and disadvantages of these surgical treatments.

Few cases are suitable for the Batista procedure; hence, a clear and reproducible technique is crucial to success. The addition of intraoperative echocardiography may provide benefit during the Batista procedure.

## CONCLUSION

Our proposed use of intraoperative direct echocardiography was useful in making the Batista procedure less technically demanding. It may be reproducible in patients with end-stage heart failure.

**Table:** 

Authors' roles & responsibilities	
KN	Substantial contributions to the conception or design of the work; or the acquisition, analysis or interpretation of data for the work; drafting the work or revising it critically for important intellectual content; final approval of the version to be published
TU	Substantial contributions to the conception or design of the work; final approval of the version to be published
AH	Agreement to be accountable for all aspects of the work in ensuring that questions related to the accuracy of integrity of any part of the work are appropriately investigated and resolved; final approval of the version to be published
MS	Final approval of the version to be published

## References

[1] Batista RJ, Verde J, Nery P, Bocchino L, Takeshita N, Bhayana JN (1997). Partial left ventriculectomy to treat end-stage heart disease. Ann Thorac Surg.

[2] Wilhelm MJ, Hammel D, Schmid C, Kröner N, Stypmann J, Rothenburger M (2005). Partial left ventriculectomy and mitral valve repair: favorable short-term results in carefully selected patients with advanced heart failure due to dilated cardiomyopathy. J Heart Lung Transplant.

[3] Isomura T, Suma H, Horii T, Sato T, Kikuchi N (2000). Partial left ventriculectomy, ventriculoplasty or valvular surgery for idiopathic dilated cardiomyopathy - the role of intra-operative echocardiography. Eur J Cardiothorac Surg.

[4] McCarthy JF, McCarthy PM, Starling RC, Smedira NG, Scalia GM, Wong J (1998). Partial left ventriculectomy and mitral valve repair for end-stage congestive heart failure. Eur J Cardiothorac Surg.

[5] Domingues JS, Vale Mde P, Barbosa MP (2015). Partial left ventriculectomy: have well-succeeded cases and innovations in the procedure been observed in the last 12 years?. Braz J Cardiovasc Surg.

[6] Isomura T, Fukada Y, Miyazaki T, Yoshida M, Morisaki A, Endo M (2017). Posterior ventricular restoration treatment for heart failure: a review, past, present and future aspects. Gen Thorac Cardiovasc Surg.

